# A Conserved Motif in the Linker Domain of STAT1 Transcription Factor Is Required for Both Recognition and Release from High-Affinity DNA-Binding Sites

**DOI:** 10.1371/journal.pone.0097633

**Published:** 2014-05-21

**Authors:** Bettina Hüntelmann, Julia Staab, Christoph Herrmann-Lingen, Thomas Meyer

**Affiliations:** Klinik für Psychosomatische Medizin und Psychotherapie, Georg-August-Universität Göttingen, Göttingen, Germany; Imperial College London, United Kingdom

## Abstract

Binding to specific palindromic sequences termed gamma-activated sites (GAS) is a hallmark of gene activation by members of the STAT (*s*ignal *t*ransducer and *a*ctivator of *t*ranscription) family of cytokine-inducible transcription factors. However, the precise molecular mechanisms involved in the signal-dependent finding of target genes by STAT dimers have not yet been very well studied. In this study, we have characterized a sequence motif in the STAT1 linker domain which is highly conserved among the seven human STAT proteins and includes surface-exposed residues in close proximity to the bound DNA. Using site-directed mutagenesis, we have demonstrated that a lysine residue in position 567 of the full-length molecule is required for GAS recognition. The substitution of alanine for this residue completely abolished both binding to high-affinity GAS elements and transcriptional activation of endogenous target genes in cells stimulated with interferon-γ (IFNγ), while the time course of transient nuclear accumulation and tyrosine phosphorylation were virtually unchanged. In contrast, two glutamic acid residues (E559 and E563) on each monomer are important for the dissociation of dimeric STAT1 from DNA and, when mutated to alanine, result in elevated levels of tyrosine-phosphorylated STAT1 as well as prolonged IFNγ-stimulated nuclear accumulation. In conclusion, our data indicate that the kinetics of signal-dependent GAS binding is determined by an array of glutamic acid residues located at the interior surface of the STAT1 dimer. These negatively charged residues appear to align the long axis of the STAT1 dimer in a position perpendicular to the DNA, thereby facilitating the interaction between lysine 567 and the phosphodiester backbone of a bound GAS element, which is a prerequisite for transient gene induction.

## Introduction

Signal-dependent transcription factors that specifically bind to DNA sequences distant from the transcriptional initiation site are required for inducible gene expression and act in concert with components of the general transcriptional machinery to recruit the DNA-dependent RNA polymerase II to transcription start sites. The *s*ignal *t*ransducers and *a*ctivators of *t*ranscription (STAT) are a well-known class of transcriptional regulatory proteins that serve the dual function of signal transduction and transcriptional activation [1]–[3]. STATs provide a crucial signalling link between complexes of ligand-bound cytokine receptors at the plasma membrane and gene transcription in the nucleus. Seven mammalian STAT genes have been identified, and their encoded proteins all function as cytokine-activated transcription factors, which play a vital role in such diverse processes as growth control, proliferation, and immune activation.

Probably best studied is the founding member of this family, STAT1, which transmits interferon signals to changes in gene expression. Cell surface binding of interferon-γ (IFNγ) induces dimerization of the two receptor subunits IFNGR1 and IFNGR2 which is followed by the auto-phosphorylation of non-covalently receptor-associated Janus kinases (JAKs). Activated JAK1 and JAK2 then phosphorylate the intracellular domain of IFNGR1, thereby creating phospho-tyrosine docking sites that recruit STAT1 proteins via their Src-homology-2-(SH2) domain to the carboxy-terminal receptor tail [4]. Subsequently, JAKs phosphorylate a signature tyrosine residue in the STAT1 carboxy-terminus (Y701) leading to the formation of phospho-STAT1 homodimers, which are stabilized through reciprocal SH2-phosphotyrosine interactions in a parallel dimer conformation [5]–[8]. Tyrosine-phosphorylated STAT1 dimers are then translocated to the nucleus via a Ran-dependent, importin-α/β-mediated transport mechanism [9]–[13]. In the nucleus, STAT1 dimers are released as import cargo and bind to palindromic gamma-activated site (GAS) elements with the consensus sequence 5′-TTC(N)_3-4_GAA-′3 in the promoter regions of IFNγ-responsive genes, where they function as cytokine-regulated transcription factors [14]–[16]. After its release from DNA, STAT1 is then dephosphorylated by the nuclear tyrosine phosphatase Tc45 [17]–[19] and exported from the nucleus to participate in another round of nucleocytoplasmic shuttling [20]. Independent of cytokine stimulation, unphosphorylated STAT molecules also translocate constitutively between the cytoplasm and the nucleus in both directions [21]. This transport pathway occurs at high exchange rates and is mediated through direct contacts of residues in the STAT1 linker domain with nucleoporins located in the nuclear pore complex [22].

While it has been well established that STAT1 is engaged in executing IFNγ-induced signal transduction [23]–[25], much less is known about the precise molecular mechanisms on how association with DNA and recruitment to GAS sequences is achieved. In this investigation, we report that a defect in the recognition of GAS sites is not necessarily linked to altered rates of nucleocytoplasmic shuttling and severely affects cytokine-induced transcriptional activation. Furthermore, we show that, in combination with a key lysine residue, two negatively charged amino acid residues located in the STAT1 linker domain allow for the spatial orientation of the DNA double helix in relation to transcriptional active STAT1 dimers which are required for GAS recognition and gene expression.

## Materials and Methods

### Cell Culture

HeLa cells expressing endogenous STAT1 and STAT1-negative U3A cells [26] were cultured at 37°C in a humidified 5% CO_2_ atmosphere in Quantum 101 medium and Dulbecco’s modified Eagle’s medium (both obtained from PAA Laboratories), respectively. For both human cell lines, media were supplemented with 10% foetal calf serum (FCS; Biochrom), 1% penicillin, and 1% streptomycin. Transfection was achieved with MegaTran1.0 (Origene) according to the manufacturer’s recommendation. Twenty-four hours after transfection, cells were either left unstimulated or stimulated with 5 ng/ml recombinant human IFNγ (Biomol). Subsequently, cells were incubated with either 500 nM staurosporine (Sigma-Aldrich) or a combination of 0.8 mM sodium vanadate and 0.2 mM H_2_O_2_ for the time periods indicated. The anti-fungal antibiotic leptomycin B (LMB, Sigma-Aldrich) was used at a final concentration of 10 ng/ml to block CRM1-mediated export.

### Plasmids

Cells were transfected with the expression vector pEGFP-N1-STAT1, termed pSTAT1, which coded for a carboxy-terminal fusion of full-length human STAT1 (amino acids 1–746) with green fluorescent protein (GFP) [27]. For transfection of untagged STAT1 in U3A cells, the expression plasmid pcDNA3.1 (Invitrogen) was used, which coded for full-length human STAT1 cDNA. DNA-binding affinity of the STAT1 mutants was probed by means of fluorescence microscopy using the construct pSTAT1-NES-GFP. This plasmid contained a sequence coding for a transferable nuclear export signal (NES) derived from STAT1 (amino acids 367–427) situated between the cDNAs for full-length STAT1 and GFP [28]. Point mutations in these three expression vectors were introduced by site-directed point mutagenesis using the QuikChange II kit from Stratagene, as recommended by the manufacturer. The following primer pairs were used (only forward primers are shown, mutated codons underlined):

E559A: 5′-CCCTTCTGGCTTTGGATTGCAAGCATCCTAGAACTCATTAAAAAAC-′3, E563A: 5′-GGATTGAAAGCATCCTAGCACTCATTAAAAAACACCTGC-′3,

K567A: 5′-CATCCTAGAACTCATTAAAGCACACCTGCTCCCTCTCTGG-′3.

All mutations were verified by standard didesoxy termination DNA sequencing (Seqlab).

### Protein Extraction

STAT1-expressing cells grown on 6-well dishes were lysed for 5 min on ice in 50 µl cytoplasmic extraction buffer (20 mM Hepes, pH 7.4, 10 mM KCl, 10% (v/v) glycerol, 1 mM EDTA, 0.1 mM Na_3_VO_4_, 0.1% IGEPAL-CA-360, 3 mM DTT, 0.4 mM Pefabloc, Complete Mini protease inhibitors (Roche)). The lysates were centrifuged at 16000 g (15 sec, 4°C), and supernatants spun again at 4°C for 5 min at 16000 g. The supernatants resulting from this centrifugation step were used as cytoplasmic extracts for Western blotting, electrophoretic mobility shift assays (EMSAs) and *in vitro* dephosphorylation assays. The pellets were resuspended in 50 µl nuclear extraction buffer (20 mM Hepes, pH 7.4, 420 mM KCl, 20% (v/v) glycerol, 1 mM EDTA, 0.1 mM Na_3_VO_4_, 3 mM DTT, 0.4 mM Pefabloc, and Complete Mini protease inhibitors) and left on ice for 30 min with occasional gentle agitation. The samples were spun at 16000 g for 15 min and 4°C and the supernatants collected as nuclear extracts. For whole cell extracts, the same amounts of cytoplasmic and nuclear extracts were mixed.

### Western Blotting

The combined cytoplasmic and nuclear lysates were boiled in SDS sample buffer and resolved by 10% SDS-PAGE with subsequent transfer onto PVDF membranes. The membranes were incubated first with a polyclonal phospho-STAT1-Tyr701-specific antibody (Cell Signaling) and then with a conjugated secondary antibody (LI-COR). To determine the amount of total STAT1, blots were stripped for 60 min at 60°C in a buffer containing 2% SDS, 0.7% β-mercaptoethanol, and 62.5 mM Tris-HCl, pH 6.8 and then reprobed with the STAT1-specific polyclonal antibody C-24 (Santa Cruz Biotechnology). Bound immunoreactivity was detected with secondary anti-rabbit IRDye 800CW antibody visualized on a LI-COR Odyssey imaging machine.

### *In vitro* Dephosphorylation Assay

*In vitro* dephosphorylation assays were performed at 30°C for 30 min with 10 µl of cellular extracts from STAT1-reconstituted U3A cells and a similar volume of dephosphorylation buffer containing 25 mM Tris-HCl, pH 7.5, 50 mM KCl, 5 mM EDTA, 4 mM dithiothreitol, 0.5 mg/ml bovine serum albumin, Complete protease inhibitors, and 2 U of the T-cell protein tyrosine phosphatase Tc45 (Biomol International). The samples contained either no DNA or duplex oligonucleotides (2xGAS or GAS-nonGAS) at a final concentration of 25 nM. Dephosphorylation reactions were stopped by adding SDS sample buffer and boiling the samples for 3 min. The amount of tyrosine-phosphorylated and total STAT1 was tested in each sample by means of Western blotting.

### Fluorescence Microscopy

To assess the kinetics of IFNγ-induced nuclear accumulation of wild-type and mutant STAT1, direct fluorescence microscopy was performed. Transiently transfected HeLa cells expressing GFP-tagged STAT1 variants were treated as indicated or left untreated. Samples were fixed in 4% paraformaldehyde in phosphate-buffered saline (PBS) for 15 min at room temperature (RT) and nuclei were stained for 10 min with 5 µg/ml Hoechst 33258 (Sigma-Aldrich). Samples were mounted in fluorescence mounting medium (Southern Biotech) and visualized using an Axiovert 200M microscope (Carl Zeiss) equipped with appropriate fluorescence filters. Images were obtained with a CCD camera and further processed with the Image-Pro MDA5.1 (Media Cybernetics) software. In each sample, STAT1-GFP fluorescence intensities were determined both in the nucleus and in the cytoplasm. The ratio of nuclear-to-total cytoplasmic fluorescence intensity was calculated, and the mean and the standard deviation depicted in a histogram.

### Immunocytochemistry

Nuclear accumulation of recombinant untagged STAT1 was immunocytochemically detected in U3A cells expressing wild-type or mutant STAT1. Adherent cells grown on 8-well chamber slides were either left untreated or treated with IFNγ for 45 min. Interferon-prestimulated cells were then incubated in the presence of 500 nM staurosporine for an additional 0, 30 or 60 min and then fixed with methanol at −20°C for 20 min. After two washes in PBS, the cells were permeabilized with 1.0% Triton X-100 in PBS for 20 min and non-specific binding was blocked by incubation with 25% FCS/PBS for 45 min at RT. The samples were incubated for 45 min with anti-STAT1 antibody C-24 (Santa Cruz) diluted 1∶1000 in 25% FCS/PBS. After three washes in PBS, the specimens were incubated with Cy3-conjugated secondary antibody (Dianova), diluted 1∶500 in PBS, for 45 min at RT followed by nuclear staining with Hoechst dye. Finally, the samples were mounted and images were captured by fluorescence microscopy. Quantification was as described above.

### Digitonization

Nuclear export of the STAT1 mutants was assessed in digitonin-permeated cells [29]. Briefly, adherent HeLa cells expressing GFP-tagged STAT1 or STAT1-reconstituted U3A cells were stimulated for 45 min with IFNγ to induce nuclear accumulation of recombinant STAT1. Then cells were either left untreated or permeabilized in the presence of 50 µg/ml digitonin (Roche) in transport buffer (0.2% Triton X-100, 10 mM KCl, 1.5 mM MgCl_2_, 10 mM Hepes, pH 7.4, 1 mM DTT, Complete protease inhibitors) for 6 min on ice. After two washes in ice-cold transport buffer, cells were fixed for 15 min at RT with 4% paraformaldehyde in PBS followed by staining with Hoechst dye. The presence of STAT1-GFP in the nuclei of HeLa cells was probed by means of direct fluorescence microscopy, while localization of untagged STAT1 was detected immunocytochemically, as described.

### Electrophoretic Mobility Shift Assay

Cellular extracts from IFNγ-stimulated cells expressing STAT1-GFP or recombinant untagged STAT1 were probed for DNA-binding activity to various duplex oligonucleotides containing consensus or mutant GAS sites [27]. Four microliters of each extract were incubated with 1 ng of the [^33^P]-labelled duplex oligonucleotide probe, generated by an end-filling reaction using the Klenow fragment (New England Biolabs). The following duplex oligonucleotides were used (4 bp overhangs at the 5′ ends and the respective antisense oligos are not included, GAS sites underlined): M67; 5′-CGACATTTCCCGTAAATCTG-′3,

2xGAS; 5′-CGTTTCCCCGAAATTGACGGATTTCCCCGAAAC-′3,

GAS-nonGAS; 5′-CGTTTCCCCGAAATTGACGGATTTACCCCAAC-′3,

2xnonGAS; 5′-CGTTTACCCCAAATTGACGGATTTACCCCAAC-′3,

CCL2 native; 5′-CTGCTTCCCTTTCCTACTTCCTGGAAATCCA-′3, and

CCL2 mutant; 5′-CTGCTTCCCTTTCCTACTAGCTGGAAATCCA-′3.

For competition experiments, cell extracts were mixed with [^33^P]-labelled duplex oligonucleotides in EMSA buffer and left for 15 min at RT. Subsequently, a 750-fold molar excess of unlabelled M67 DNA was added and incubated for the indicated times on ice [30]. In supershift assays, 20 ng of either the STAT1-specific antibody C-24 or a non-specific STAT3 antibody were preincubated with the shift reaction for 15 min at RT. The reactions were loaded on a 4% 29∶1 acrylamide:bisacrylamide gel at 4°C and separated at 400 V. STAT1 DNA-binding activity was visualized on vacuum-dried gels with a phosphoimaging system (FLA-5100, Fuji) using the programs Aida Image Analyzer v. 4.06 and TINA 2.0 (Raytest).

### Pull-down Assay with Biotinylated Oligos

Biotin-modified duplex oligonucleotides (0.5 ml, 25 pmol/µl) containing either two optimal GAS sites (2xGAS) or a permutated sequence thereof (2xnonGAS) were conjugated to streptavidin agarose (0.5 ml packed vol., Sigma-Aldrich) for 1 h at 4°C. Confluent U3A cells (one 10-cm dish) transiently expressing recombinant, untagged STAT1 were either left untreated or stimulated for 30 min with IFNγ and 15 min in the additional presence of vanadate/H_2_O_2_ before being lysed in a total volume of 400 µl extraction buffer, as described above. After pre-clearing with streptavidin agarose, 320 µl of the extracts were rotated with 50 µl (packed vol.) of DNA-conjugated beads for 2 h at 4°C. The beads were washed with cytoplasmic extraction buffer (400 µl), bound proteins were eluted by boiling in SDS sample buffer and analyzed by Western blotting.

### Reporter Gene Assay

U3A cells grown on 48-well plates were co-transfected in each well with the following three plasmids: 70 ng of a luciferase reporter, 200 ng of a constitutively expressed β-galactosidase plasmid, and 250 ng of a pSTAT1 plasmid encoding either the wild-type protein or one of the three mutants under investigation. The reporter gene contained a triple Ly6E STAT-binding site (termed 3xLy6E) upstream from the transcription start site [31]. Twenty-four hours post-transfection, cells were either left untreated or treated for 6 h with IFNγ, before whole cell extracts were prepared with a lysis buffer containing 25 mM glycylglycine, 1% Triton X-100, 15 mM MgSO_4_, 4 mM EGTA, 0.4 mM Pefabloc (Sigma-Aldrich), 3 mM DTT, pH 7.8, and Complete protease inhibitors. In each sample, luciferase expression was assessed (Promega) using the luminometer Centro KS LB960 (Berthold Technologies) and the software program MikroWin. Luciferase expression was normalized to the corresponding β-galactosidase activity, which was measured spectroscopically at 420 nm. For each STAT1 variant and stimulation mode, six independent transfections were tested and the experiment was repeated at least in triplicate.

### Real-time PCR

U3A cells transfected with pcDNA3.1 expression plasmids coding for wild-type or mutant STAT1 were starved for 15 h in Dulbecco’s modified Eagle’s medium supplemented with 1% FCS. Cells were then either left untreated or stimulated for 6 h with IFNγ. RNA was isolated with the peqGold Total RNA kit (Peqlab), and first-strand cDNA synthesis was performed using the Verso cDNA Synthesis kit from Thermo Fisher Scientific. The real-time PCR reactions were carried out in a total volume of 20 µl, containing 25 ng cDNA, 70 nmol/l of each specific primer pair, and 10 µl Absolute Blue SYBR Green Mix (Thermo Fisher Scientific). The following primer pairs were used: GBP1F, 5′-GGTCCAGTTGCTGAAAGAGC-′3; GBP1R, 5′-TGACAGGAAGGCTCTGGTCT-′3; IRF1F, 5′-AGCTCAGCTGTGCGAGTGTA-′3;

IRF1R, 5′-TAGCTGCTGTGGTCATCAGG-′3; MIG1F, 5′-CCACCGAGATCCTTATCGAA-′3; MIG1R, 5′-CTAACCGACTTGGCTGCTTC-′3; CCL2F, 5′-CCAGTCACCTGCTGTTATAAC-′3, CCL2R, 5′-TGGAATCCTGAACCCACTTCT-′3; GAPDHF, 5′-GAAGGTGAAGGTCGGAGTC-′3; GAPDHR, 5′-GAAGATGGTGATGGGATTTC-′3; STAT1F, 5′-CCGTTTTCATGACCTCCTGT-′3; and STAT1R, 5′-TGAATATTCCCCGACTGAGC-′3. The following PCR protocol run on an Eppendorf cycler was applied: a denaturation step at 95°C for 15 min and 40 cycles of denaturation at 95°C for 15 s, annealing at 55°C for 30 s, and extension at 72°C for 30 s. After the final amplification step, a melting curve analysis was run via a temperature gradient from 60°C to 95°C in 0.5°C increment steps, fluorescence being measured at each temperature for a period of 10 s. All reactions were carried out in at least triplicate for each sample. The relative expression of a transcript was normalized to the expression of *gapdh* as determined for each sample. The ΔΔCt-method was used to determine comparative relative expression levels, based on the formula 2^-(ΔCt target - ΔCt reference sample)^ [32].

### Statistical Analyses

Means and standard deviations were calculated for each variant and stimulation mode. Differences in DNA-binding activity, gene expression and nucleocytoplasmic localization among the STAT1 variants were assessed using Student’s *t* tests and Mann-Whitney-Wilcoxon tests, where appropriate. Data from reporter gene assays were analyzed by ANOVA with Tukey’s post-hoc tests. All data were analyzed using the Sigmastat (Systat Software) program. In all analyses, a p value ≤0.05 was used to indicate statistical significance.

## Results

### Identification of a Conserved Sequence Motif in the STAT1 Linker Domain

**Figure 1:**
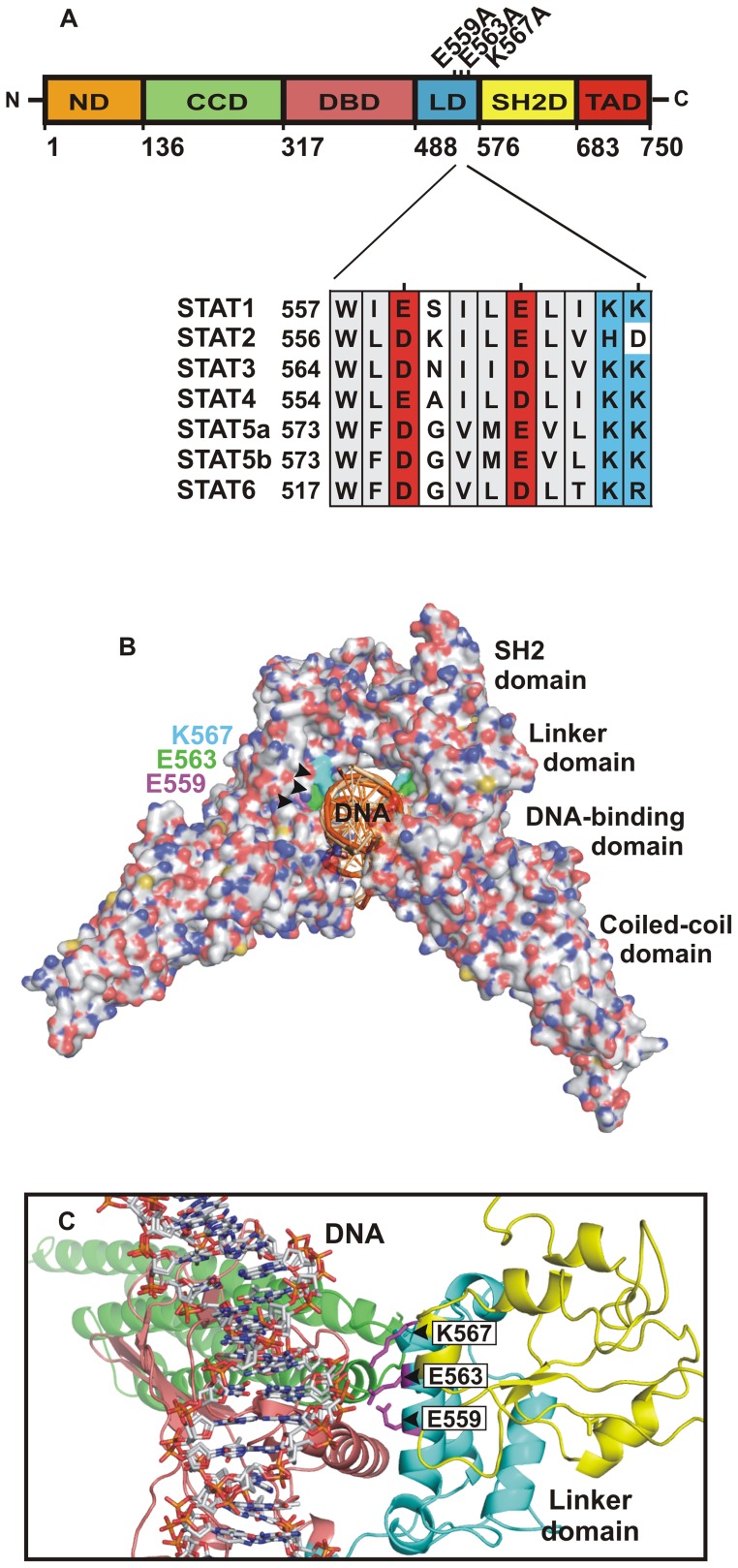
Localization of the two glutamic acid residues 559 and 563 and lysine 567 in the STAT1 linker domain. (A) Modular domain structure of STAT1 and sequence alignment of the three residues E559, E563, and K567 under investigation. The schematic diagram depicts the domain boundaries of the STAT1 protein including the N-domain (ND), the coiled-coil domain (CCD), the DNA-binding domain (DBD), the linker domain (LD), the SH2 domain (SH2D), and the transactivating domain (TAD). (B) Crystal structure of the DNA-bound truncated STAT1 dimer [43] showing the spatial orientation of the side chains of E559 (coloured in magenta), E563 (green), and K567 (cyan) which are all located in the groove formed between the STAT1 linker domain and DNA. (C) Close-up view of a ribbon diagram of STAT1 confirms that the three residues indicated have free access to the DNA double helix. Figures B and C were prepared using the program PyMOL (DeLano Scientific).

A consensus motif was identified in the linker domain of the seven human STAT proteins which contains the undecapeptide structure W-Ψ-D/E-x-Ψ-Ψ-D/E-Ψ-Ψ-K/H-K/D/R, where Ψ is a hydrophobic residue, x any residue, D or E residues with negatively charged and K, H or R with positively charged side chains, respectively (Fig. 1A). In all human STAT family members there is either an aspartic or glutamic acid residue in positions 3 and 7 of this consensus motif, while in position 11 lysine or arginine (in STAT6) is positioned, except for STAT2 where aspartic acid is located in this position. Structural data of the DNA-bound STAT1 dimer demonstrated that the terminal carboxyl group of E563 lies at a distance of 3.7 Å from the phosphodiester backbone of the co-crystallized DNA double helix. The side chain of the K567 residue is 3.4 Å away from the DNA backbone without any other residue in the STAT1 molecule to prevent its free access to DNA. All three residues are surface- exposed on a single α-helix in the linker domain, which runs almost parallel to the longitudinal axis of the DNA molecule, with their side chains in close proximity to the double helix (Fig. 1B). The terminal amino group of K567 is located most medially in a ring-like groove that is formed anteriorly by the DNA and posteriorly by the two STAT1 partner monomers bound to each other via reciprocal SH2-phosphotyrosine interactions (Fig. 1C).

### Prolonged Cytokine-induced Nuclear Accumulation of STAT1-E559A and -E563A

**Figure 2:**
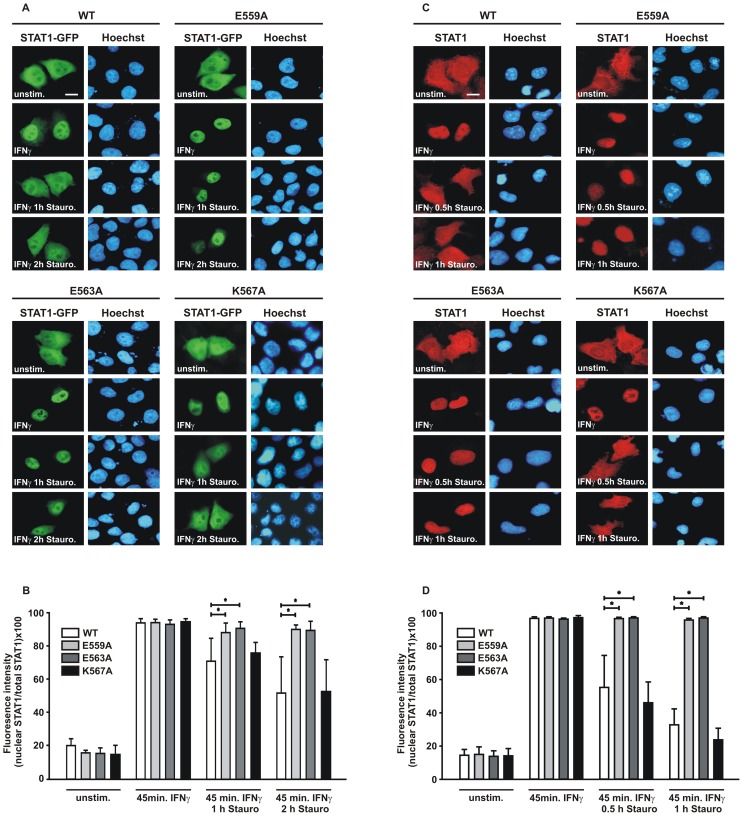
Replacement of glutamine residues in position 559 and 563 results in STAT1 mutants with a prolonged IFNγ-induced nuclear accumulation. (A, B) HeLa cells expressing GFP fusion proteins with either STAT1-WT, -E559A, -E563A, or -K567A were untreated or stimulated for 45 min with 5 ng/ml human IFNγ followed by exposure to staurosporine (500 nM) for 0 min, 60 min and 120 min, respectively. The fluorescence micrographs show the intracellular distribution of the GFP-tagged fusion proteins and the localization of the corresponding Hoechst-stained nuclei (scale bar 10 µm). (B) Quantification of the nucleocytoplasmic STAT1-GFP distribution in untreated and IFNγ-pretreated cells exposed to staurosporine as determined from experiment (A). The histograms demonstrate the ratio of nuclear-to-total cellular fluorescence intensities as measured for the indicated STAT1 variants and stimulation modes. (C) Indirect fluorescence microscopy confirming the decreased nuclear export rates of STAT1-E559A and -E563A. STAT1-negative U3A cells, reconstituted with untagged wild-type or mutant STAT1, were treated as above, except that exposure times to staurosporine were shortened (0 min, 30 min, and 60 min). Intracellular distribution of recombinant STAT1 in the fixed, Hoechst-stained cells was monitored immunocytochemically using anti-STAT1 C-24 and Cy3-labelled secondary antibodies (scale bar 10 µm). (D) Nucleocytoplasmic distribution of the STAT1 mutants, as quantified from Figure 2C, with bars and asterisks indicating significant differences between wild-type and the respective mutant.

In an effort to decipher the role of this highly conserved α-helical stretch adjacent to the DNA, we took a mutational approach by substituting alanine for residues with charged side chains. The resulting mutants E559A, E563A and K567A were all normally expressed in transfected human cell lines and showed an unaltered nucleocytoplasmic distribution in the absence of cytokine stimulation similar to the wild-type protein. This was observed for fusions with green fluorescent protein (GFP) in HeLa cells (Fig. 2A, B) as well as for recombinant untagged protein in reconstituted STAT1-negative U3A cells, which lack endogenous STAT1 expression (Fig. 2C, D). Upon stimulation of cells with IFNγ, all STAT1 variants accumulated regularly in the nucleus within 45 min of the addition of the cytokine, excluding the possibility that the introduced mutations were associated with severe structural instability. Exposure of IFNγ-pretreated cells to the potent kinase inhibitor staurosporine at a concentration of 500 nM led to the reversal of STAT1 nuclear accumulation in cells expressing the wild-type molecule. While for wild-type STAT1 the nearly pancellular localization in resting cells was restored already 60 min after staurosporine treatment, the cytoplasmic concentration of the two glutamic acid-to-alanine mutants was still much lower than in the nucleus. Even after 2 hours of staurosporine treatment there was evidence of a diminished nuclear export of the two mutants, as suggested by their prolonged nuclear accumulation phase. However, we found no difference in the kinetics of nuclear export between wild-type STAT1 and the K567A mutant, irrespective of whether the GFP fusions (Fig. 2A, B) or the untagged STAT1 was tested (Fig. 2C, D).

### Mutation of Two Glutamine Acid Residues in the Linker Domain Results in Increased Tyrosine Phosphorylation

**Figure 3:**
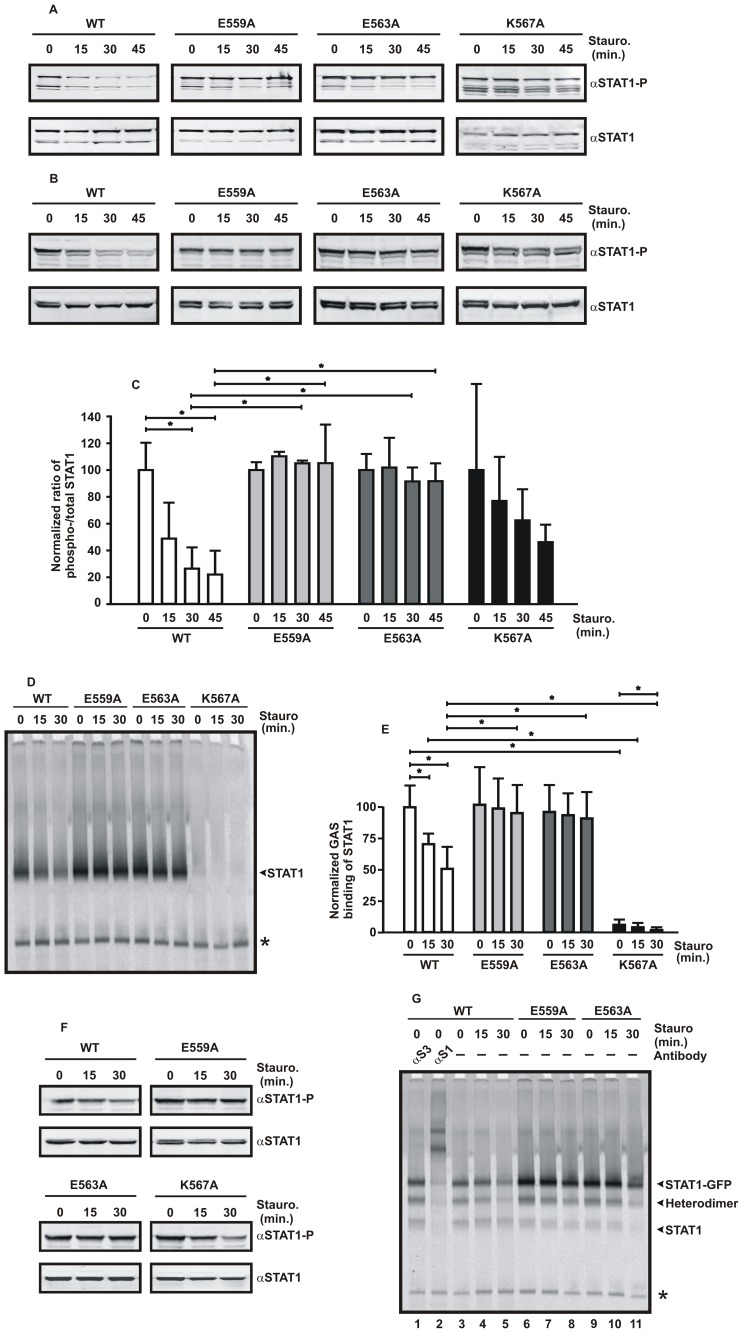
(A) Hyperphosphorylation of the STAT1 mutants E559A and E563A upon stimulation of cells with IFNγ. Equal cell numbers of HeLa cells expressing either wild-type or one of the indicated STAT1 mutants, all fused to GFP, were prestimulated for 45 min with 5 ng/ml IFNγand subsequently exposed to the kinase inhibitor staurosporine for increasing time periods, before tyrosine phosphorylation was tested for in cell lysates. A representative Western blot experiment using a STAT1-specific phospho-tyrosine antibody (top panel) and the corresponding re-blot after the stripping off of bound immunoreactivity and re-incubation with pan-STAT1 antibody C-24 (bottom panel) are shown. The upper band on each blot marks recombinant STAT1-GFP, whereas the lower band corresponds to endogenous STAT1. (B) Elevated and prolonged tyrosine phosphorylation levels of the E559A and E563A mutants in STAT1-reconstituted U3A cells. Similar experiment as in Figure 3A, except that U3A cells were transfected with the respective pSTAT1-GFP constructs. Staurosporine treatment resulted in the rapid loss of tyrosine-phosphorylated STAT1-WT and -K567A, whereas the two glutamic acid mutants partially resisted the inactivating effect of the kinase blocker. (C) Quantification of immunoblots for expression of tyrosine-phosphorylated and total STAT1 in IFNγ-pretreated (45 min) U3A cells exposed to staurosporine for different time periods, as in Figure 3B. Significant differences in the ratio of phosphorylated to total STAT1 between different incubation times or STAT1 variants are marked with bars and asterisks. (D) Prolonged GAS-binding activity of tyrosine-phosphorylated E559A and E563A and defective binding of the phospho-K567A mutant. Gel shift experiment with cellular extracts from STAT1-reconstituted U3A cells treated as described above. Cell lysates were equilibrated with a radioactively labelled high-affinity STAT-binding probe termed M67, before being loaded onto a non-denaturing gel. (E) GAS-binding activity from IFNγ-prestimulated U3A cells exposed to staurosporine was densitometrically analyzed. (F) The same extracts used for the EMSA experiment shown in Figure 3D were probed for the presence of phosphorylated and total STAT1. (G) Similar experiment as in Figure 3D, except that STAT1-GFP-expressing HeLa cells were used and supershifts using an unspecific STAT3-antibody (αS3, lane 1) and a specific STAT1-antibody (αS1, lane 2) were included. At the right-hand margin of the gel the positions of the GFP-tagged and untagged STAT1 homodimers as well as the corresponding STAT1-GFP/STAT1 heterodimer are indicated. Asterisks mark unspecific bands.

Given the prolonged nuclear accumulation of the two glutamic acid-to-alanine mutants, we next examined their kinetics of tyrosine dephosphorylation in IFNγ-stimulated cells by means of Western blotting using a phospho-STAT1-specific antibody (Fig. 3A–C). In line with the fluorescence microscopic data, we found that the ratio of tyrosine-phosphorylated STAT1 to the total intracellular STAT1 pool, which also contained unphosphorylated protein, was elevated for the E559A and E563A mutants as compared to wild-type STAT1. Staurosporine treatment in IFNγ-prestimulated HeLa cells resulted in increased tyrosine-phosphorylation of the two GFP-tagged mutants as compared to the wild-type molecule (Fig. 3A). Similar results were observed for the untagged STAT1 variants in reconstituted U3A cells (Fig. 3B, C). Elevated tyrosine-phosphorylation levels were also detected in IFNγ-stimulated cells in the absence of staurosporine treatment (Fig. S1). However, there was no significant difference between wild-type STAT1 and the substitution mutant K567A (Fig. 3C).

Gel shift assays confirmed the results from the Western-blot experiments by demonstrating an increased DNA-binding activity of the two glutamic acid-to-alanine mutants (Fig. 3D–G). Exposure of IFNγ-pretreated U3A cells to staurosporine resulted in a rapid dephosphorylation of the wild-type protein already within 15 min, while the E559A and E563A mutants exhibited a much lower dephosphorylation rate (Fig. 3D–F). However, virtually no binding to the radioactively labelled GAS probe was visualized for the K567A mutant (Fig. 3D, E), although immunoblotting experiments using the same extracts showed unaltered expression of the tyrosine-phosphorylated molecule (Fig. 3F). In HeLa cells expressing GFP-fusion proteins, increased M67 binding was detected not only for homodimers of STAT1-E559A and -E563A, but also for heterodimers formed between recombinant and endogenous STAT1 monomers (Fig. 3G).

### Substitutions in the Linker Domain Differentially Affect DNA Binding

**Figure 4:**
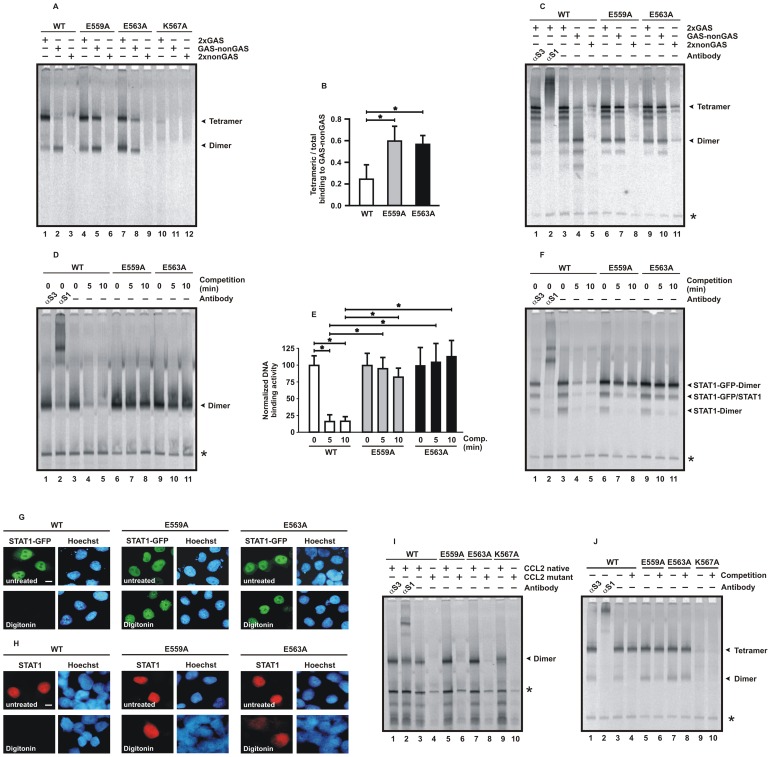
The point mutants E559A, E563A and K567A display altered DNA-binding kinetics. (A–C) Binding affinity of the indicated STAT1-GFP fusion proteins to three different duplex oligonucleotides containing two strong GAS elements in tandem arrangement (2xGAS) or mutations thereof, with a single GAS element (GAS-nonGAS) or no GAS element (2xnonGAS). The images depict representative EMSA results using whole cell extracts from reconstituted U3A cells (A) and HeLa cells (C) with the positions of tetrameric and dimeric GFP-tagged STAT1 indicated at the right-hand margin of the gel. Where indicated, anti-STAT3- (αS3) and anti-STAT1-antibodies (αS1) were included in the reactions. The additional bands in HeLa extracts correspond to homo- and heterodimers of recombinant GFP-tagged STAT1 as well as homo- and heterotetramers of native STAT1 lacking the GFP fusion. (B) The percentage of tetrameric-to-total STAT1 complexed to GAS-nonGAS was significantly elevated in the case of glutamic acid point mutants as compared to the wild-type protein. (D) Mutation of either glutamic acid 559 or 563 to alanines results in decreased dissociation rates from DNA and high-affinity GAS binding. Whole cell extracts from STAT1-GFP-reconstituted U3A cells were incubated for 15 min with [^33^P]-labelled DNA containing a single STAT binding site (M67) and, subsequently, a 750-fold molar excess of unlabelled DNA was added for 0, 5 and 10 min, respectively, before the samples were loaded onto a native polyacrylamide gel. In the second lane, anti-STAT1 antibody C-24 was present in the EMSA reaction for the identification of STAT1-M67 complexes which are marked with an arrowhead. (E) Dissociation of STAT1-GFP from M67 was analyzed quantitatively confirming the high stability of DNA-bound mutant STAT1 in comparison to wild-type STAT1. (F) Similar EMSA experiment as in Figure 4D, except that STAT1-GFP-expressing HeLa cells were used. Note that not only STAT1-GFP homodimers but also heterodimers of recombinant and native STAT1 are partially protected from competition. (G, H) Reduced nuclear export kinetics of STAT1-E559A and -E563A. HeLa cells expressing GFP-tagged wild-type or mutant STAT1 (scale bar 10 µm) were prestimulated for 45 min with IFNγ to induce nuclear accumulation (top panel) and then treated for 6 min in the presence of 50 µg/ml digitonin in ice-cold transport buffer (bottom panel). Fluorescence micrographs of formaldehyde-fixed cells demonstrating the amount of nuclear STAT1-GFP and the localization of the corresponding Hoechst-stained nuclei before and after digitonin treatment are shown in (G). (H) Similar experiment as in (G), except that immunofluorescence staining of transfected U3A cells was performed. (I) STAT1-K567A is a DNA-binding mutant. DNA binding activity to a [^33^P]-labelled native *ccl2* promoter element (CCL2 native), but not to a mutated version thereof (CCL2 mutant), was detected in extracts from reconstituted U3A cells by means of EMSA. (J) Weak binding of tetrameric STAT1-K567A to [^33^P]-labelled DNA containing a tandem GAS site. The reactions were either left unchallenged (−) or challenged for 20 min with a 750-fold molar excess of a single, unlabelled GAS site (+ competition). At the margin of the EMSA gel, the positions of tetrameric (top arrowhead) and dimeric (bottom arrowhead) STAT1 are marked. Asterisks label a non-specific band.

Next, we tested our mutants for binding to DNA probes containing either two classical GAS sites in tandem orientation (termed 2xGAS) or a mutated sequence thereof with only one (GAS-nonGAS) and no intact GAS site (2xnonGAS), respectively. Electrophoretic mobility shift assays (EMSA) showed that for STAT1-WT, -E559A, and -E563A the amount of 2xGAS complexed with tetrameric STAT1 was higher than DNA complexes occupied by only one dimer (Fig. 4A–C). In contrast, binding to 2xnonGAS was below the detection threshold for all variants tested. However, the ratio of tetrameric-to-dimeric STAT1 complexes bound to GAS-nonGAS differed significantly between the wild-type protein on the one hand and the glutamic acid-to-alanine mutants on the other. In U3A cells expressing untagged STAT1 (Fig. 4A, B) and in HeLa cells expressing GFP-tagged STAT1 (Fig. 4C), the amount of STAT1-WT tetramers bound to GAS-nonGAS was lower than for STAT1 dimers (Fig. 4A–C). In contrast, the two glutamic acid-to-alanine mutants more frequently occupied the GAS-nonGAS probe as tetramers than as dimers, which most likely reflected their increased concentration of tyrosine-phosphorylated molecules in the EMSA reactions as compared to the wild-type protein.

Competition gel shifts were performed to assess the dissociation rate of the substitution mutants from high-affinity DNA-binding sites (Fig. 4D–F). Extracts from reconstituted U3A cells were incubated for 15 min with a [^33^P]-labelled M67 probe, before a 750-fold molar excess of unlabelled M67 was added and incubated on ice for up to 10 min. The reactions were finally loaded onto a non-denaturing polyacrylamide gel and DNA-binding activity was assessed by means of autoradiography. Results showed a significantly reduced dissociation rate of the E559A and E563A mutants as compared to wild-type STAT1. While already 10 min after addition of unlabelled M67 duplex oligonucleotides, binding of STAT1-WT to the labelled probe was hardly detectable, the two mutant STAT1 proteins still resisted displacement by the high molar excess of unlabelled GAS sites. Thus, exchange of either of the two conserved glutamic acid residues in position 559 or 563 of the full-length molecule resulted in mutant proteins with a reduced off-rate from GAS sites.

To confirm the altered DNA-binding properties of the two mutants, we next performed a permeabilized cell transport assay using HeLa cells expressing GFP-tagged wild-type STAT1 or the respective glutamic acid mutants (Fig. 4G). The assay exploited the fact that treatment of cells with digitonin at a concentration of 50 µg/ml selectively permeabilizes the plasma membrane and, thereby, releases cytoplasmic proteins, while the integrity of the nuclear envelope remains intact [29]. The cells were first stimulated for 45 min with IFNγ to induce nuclear accumulation of the recombinant GFP fusion proteins and then incubated for 6 min on ice with or without digitonin in transport buffer. Direct fluorescence microscopy in formaldehyde-fixed cells showed that the pre-existing nuclear presence of STAT1-WT-GFP was completely abrogated through permeabilization by digitonin, while the two mutants remained accumulated in the nucleus (Fig. 4G, bottom panel). Similar results were obtained when IFNγ-prestimulated U3A cells expressing untagged STAT1 were exposed to digitonin, before being stained for indirect fluorescence microscopy using a STAT1-specific antibody (Fig. 4H). Collectively, these experiments demonstrated that the nuclear export rate was critically reduced by exchange of either of the two glutamic acid residues.

We then assessed the low GAS-binding affinity of the lysine-to-alanine mutant at position 567 in more detail. Figure 4I demonstrates a weak binding of K567A to a 32bp-fragment from the promoter of the *ccl2* gene encoding C-C chemokine motif ligand 2/monocyte chemoattractant protein 1 (termed here CCL2 native). This observation confirmed that the mutant retains a reduced affinity to GAS sites and that recognition of GAS elements is not completely abolished. The *ccl2* promoter contains a classical GAS element with an adjacent TCC motif located 10 bp upstream, and mutation of the GAS site prevented STAT1 from binding to this probe in EMSA reactions (Fig. 4I, probe termed CCL2 mutant). In addition, we detected a weak binding of tetrameric STAT1-K567A to an oligonucleotide probe with two GAS sites (Fig. 4A, J).

### Diminished Nuclear Export of Phosphorylated STAT1-E559A and -E563A

**Figure 5:**
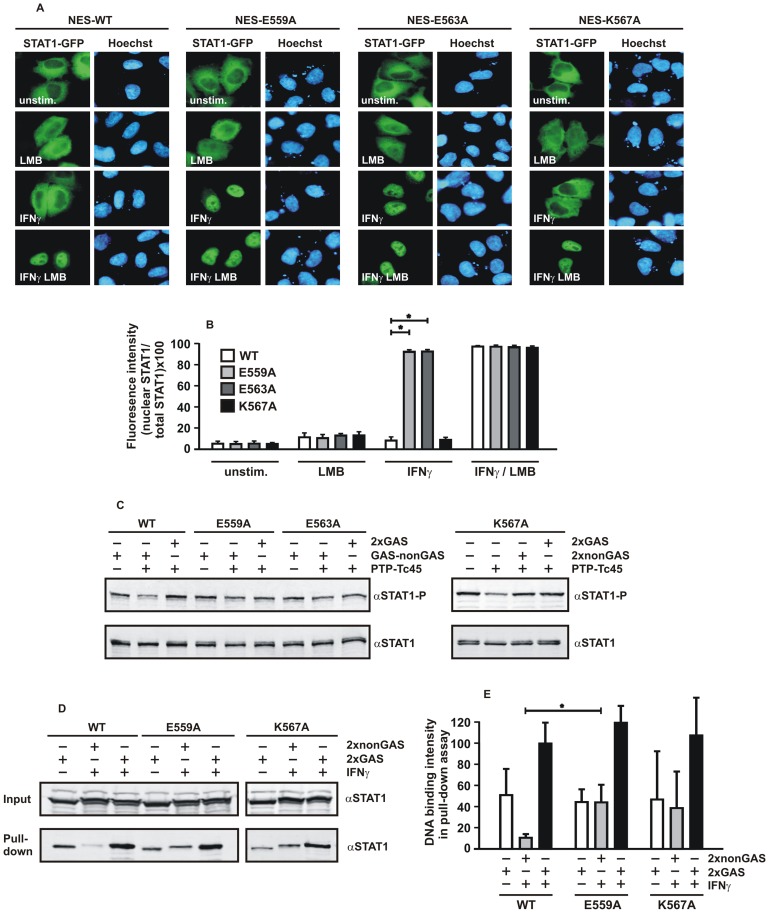
(A, B) The E559A and E563A mutants display high-affinity DNA binding and restore defective nuclear accumulation of a STAT1 variant with artificially enhanced nuclear export. HeLa cells transfected with pSTAT1-NES-GFP, which coded for a transferable nuclear export signal (NES) situated between the cDNAs for full-length STAT1 and GFP, were either left untreated or treated for 45 min with IFNγ (5 ng/ml), the CRM1 export inhibitor leptomycin B (LMB, 10 ng/ml) or a combination of the two. (A) The micrographs demonstrate the intracellular distribution of STAT1-NES-GFP and the localization of corresponding Hoechst-stained nuclei (scale bar 10 µm). (B) Histograms demonstrate the percentage of nuclear-to-total STAT1 fluorescence intensities for the various STAT1-NES proteins and stimulation modes. (C) *In vitro* dephosphorylation assays showing that DNA-bound E559A, E563A and K567A are protected from Tc45-catalyzed inactivation. Whole cell extracts from reconstituted U3A cells expressing wild-type or mutant STAT1 were incubated for 30 min with or without the recombinant Tc45 phosphatase in the absence or presence of 2xGAS, GAS-nonGAS or 2xnonGAS, as indicated. Reactions were probed for phospho-STAT1 levels by means of Western blotting (αSTAT1-P). The membrane was then stripped and re-incubated with the pan-STAT1 antibody C-24 (αSTAT1). (D) Precipitation of STAT1-WT, -E559A and -K567A with agarose-bound duplex oligonucleotides comprising tandem M67 binding sites (2xGAS), a GAS and non-GAS site (GAS-nonGAS) or a mutated sequence thereof (2xnonGAS). Beads were incubated with cell extracts from transfected U3A cells with and without IFNγ treatment. Five percent (vol/vol) of the cell extract input was blotted and probed with anti-STAT1 antibody. The precipitate (pull down) was analyzed by Western blotting with anti-Stat1 antibody. (E) Quantification of three independent pull-down experiments for each of the STAT1 variants tested, as shown in D. Significant differences between the three variants are indicated with an asterisk.

The preceding experiments had indicated that point mutants in the STAT1 linker domain exhibited unique phenotypes depending on which of the residues was replaced by alanine. To confirm that the observed properties of the mutants were a consequence of their altered DNA-binding affinity, we performed direct fluorescence microscopic studies by adding a nuclear export signal (NES) to GFP-tagged STAT1 [28] and, in another set of experiments, exposed our mutants to the STAT1-inactivating protein tyrosine phosphatase Tc45 in the presence of various DNA probes. In our first experimental approach, we introduced the mutations in a STAT1 full-length construct containing a transferable NES activity from STAT1 amino acids 367–427, which was inserted between the cDNAs for STAT1 and GFP and, after transfection in HeLa cells, determined the nucleocytoplasmic distribution of the corresponding fusion proteins (Fig. 5A, B). Due to the artificially elevated nuclear export of all NES adducts, the nuclei of unstimulated cells were virtually devoid of GFP fluorescence, while in cells treated with the exportin blocker leptomycin B (LMB) the nearly pancellular resting distribution observed for STAT1-GFP was restored. Most notably, stimulation of cells with IFNγ alone did not change the cytoplasmic localization of the NES derivatives of wild-type STAT1 and the K567A mutant, respectively, but induced a sustained nuclear accumulation of the two glutamic acid-to-alanine mutants. However, inhibition of the NES by adding LMB simultaneously with IFNγ to the cells completely restored the defective nuclear build-up of wild-type and STAT1-K567A. This observation clearly corroborated the fact that high-affinity DNA-binding is the underlying phenotype of the two glutamic acid-to-alanine mutants.

Since DNA-bound STAT1 is protected from being dephosphorylated by the STAT1-inactivating protein tyrosine phosphatase Tc45 [20], we further characterized our mutants in *in vitro* dephosphorylation assays using recombinant Tc45 and whole cell extracts in the absence or presence of DNA (Fig. 5C). While the high-affinity tandem GAS sites in 2xGAS protected all STAT1 variants from being inactivated by the phosphatase, co-incubation with lesser-affine GAS-nonGAS resulted in a reduced dephosphorylation of E559A and E563A as compared to the wild-type molecule. This observation is in line with the sequence requirements for binding to DNA, as determined from the EMSA experiments shown in Figures 4A–C. Likewise, the dephosphorylation rate of the K567A mutant was decreased when DNA was present in the reaction, irrespective of whether or not the DNA contained a functional GAS site (Fig. 5D). Densitometric analysis of pull-down assays using biotinylated duplex oligonucleotides bound to streptavidin agarose confirmed that E559A bound to unspecific DNA (2xnonGAS) 4-fold better than the wild-type protein (Fig. 5E). Similar findings were obtained for the K567A mutant and, in addition, there was no statistically significant difference in its binding affinity between specific and unspecific DNA. Together, these experiments showed that the two glutamic acid-to-alanine mutants bound DNA with increased affinity and that, through the substitution of alanine for lysine, the resulting K567A mutant had almost completely lost its discrimination for the GAS site, while unspecific DNA binding was insignificantly increased.

### DNA-binding Mutants Exhibit Distinct Gene-specific Transcriptional Responses

**Figure 6:**
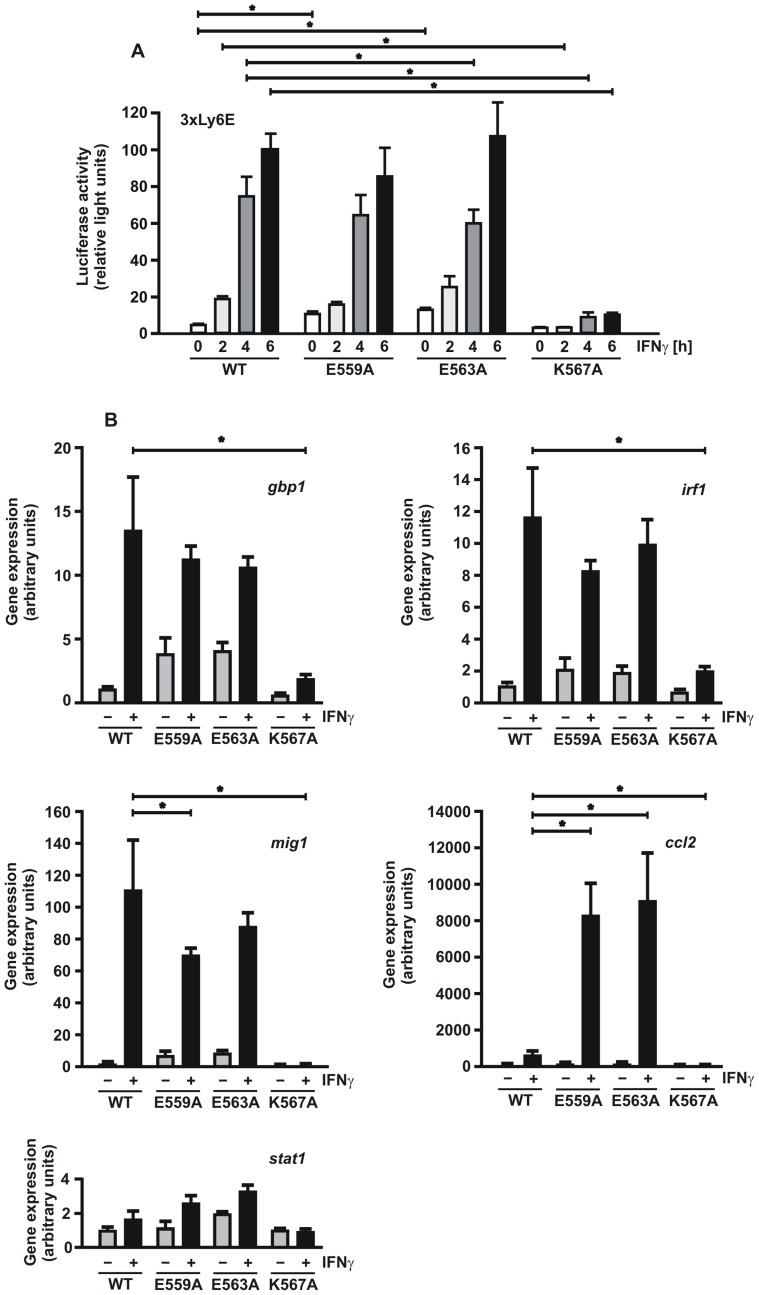
Gene-specific expression pattern of STAT1-E559A and -E563A and lack of transcriptional activation by the K567A mutant. (A) Luciferase reporter gene assay in U3A cells expressing the indicated STAT1 variants normalized to the expression of constitutively co-expressed β-galactosidase. The reporter constructs used in this experiment contained a triple GAS site from the Ly6E promoter (3xLy6E) in the luciferase gene. Cells were treated for 0 h (white columns), 2 h (bright grey), 4 h (dark grey) and 6 h (black) with 5 ng/ml IFNγ before, in whole cell extracts, luciferase luminescence and β-galactosidase activity were measured. The experiment was repeated at least three times in six independent transfections. (B) Endogenous gene induction by the STAT1 mutants was determined by real-time PCR assays. Histograms depict expression levels of the *gbp1*, *irf1*, *mig1*, *ccl2* and for control *stat1* gene before (grey columns) and after 6 h stimulation with 5 ng/ml IFNγ (black columns). Specific gene induction was normalized to the expression of the house-keeping gene *gapdh*. The data are presented as means and standard deviations from at least three independent experiments. Statistical significance between the groups of IFNγ-stimulated cells expressing the indicated STAT1 variants is marked by asterisks.

The experiments presented thus far have shown that mutations of three residues in the linker domain differentially modulate the DNA-binding kinetics of STAT1. Thus, in a final set of experiments, we compared the transcriptional activity of the respective mutants with the wild-type protein. As expected, a mutant with defective GAS recognition such as K567A failed to show any IFNγ-induced gene expression, as tested in both a luciferase reporter gene assay (Fig. 6A) and real-time PCR for four native STAT1-regulated target genes (Fig. 6B). However, DNA-binding mutants with increased affinity to GAS sites (E559A and E563A) displayed an unexpected and distinct pattern of cytokine-regulated gene activation. We found that the exchange of the two glutamic acid residues in the linker domain had no impact on the IFNγ-induced activation of the *gbp1* and *irf1* gene. However, expression of the *mig1* gene was slightly decreased by the two hyper-phosphorylated STAT1 mutants as compared to the wild-type protein. In contrast, the expression rate of the *ccl2* gene was much higher in cells expressing the glutamic acid-to-alanine mutants.

## Discussion

In the present study, we introduced three point mutations in the STAT1 linker domain and identified the phenotype of the respective mutants as being caused by altered kinetics of DNA binding. Two of the mutants under investigation, both of which were substitutions of alanine for glutamic acid residues (E559A and E563A), displayed persistent phosphorylation *in vivo* and abnormal phosphatase resistance when bound to DNA *in vitro*. Using competition gel shift experiments, GAS-bound mutant STAT1 dimers resisted challenge with excess unlabelled M67, while wild-type STAT1 showed the well-established high dissociation rate from a single GAS site, suggesting that increased affinity to DNA is the underlying molecular mechanism of these mutants. The high DNA-binding affinity may result from an increased stability of dimers when complexed with DNA which, however, cannot be resolved in EMSA assays due to the generally high off-rate from single binding sites. As expected, the DNA-binding mutants showed an impaired nuclear export of phosphorylated STAT1 dimers, which resulted from their elevated levels of tyrosine phosphorylation and, consequently, led to prolonged nuclear accumulation. In this respect, our mutations in the linker domain exhibited just the opposite phenotype to that described by Darnell and co-worker for their double mutant K544A/E545A, which has a substantially reduced residence time on STAT binding sites [33]. However, when we studied the transcriptional responses caused by our glutamic acid-to-alanine substitutions, we unexpectedly found a gene-specific activation profile instead of a global up-regulation of IFNγ-responsive target genes. While activation of two IFNγ-driven target genes (*gbp1* and *irf1*) was unaffected and expression of *mig1* was even slightly reduced by the amino acid exchanges, the mutants nonetheless induced the *ccl2* gene with a much higher efficacy, as demonstrated by real-time PCR. This complex pattern of gene activation has previously been reported for two disease-associated dimer interface mutations localized in either the coiled-coil domain (F172W) or the DNA-binding domain (T385A) [34], both of which are assumed to critically impair the conformational transition from a DNA-bound parallel to an antiparallel dimer configuration [35]–[38]. Thus, irrespective of whether high affinity binding to DNA or a hindered conformational dimer shift is causing resistance to the inactivating phosphatase, the hyperphosphorylated mutants nonetheless display a common gene expression profile.

Our transcriptional data demonstrate that the activation of the *ccl2* gene is much more sensitive to increases in the intracellular concentration of tyrosine-phosphorylated STAT1 than other genes tested, such as *gbp1* and *irf1*. The promoter of the *ccl2* gene contains “one-and-a-half” GAS sites, and due to the presence of a 5′-TTC-′3 motif at a distance of 10bp upstream from the classical GAS element, the gene appears to be particularly prone to cooperative DNA binding. Cooperative DNA binding requires two adjacent STAT1 dimers that form a tetrameric tether on DNA stabilized through N-domain-mediated interactions [39]–[42]. Our EMSA experiments showed that the mutants had a broader sequence preference, a decreased dissociation rate from DNA, and a preference for forming tetrameric complexes on DNA. In line with these observations, we found that the ratio of DNA-bound to non-bound molecules is higher for the two glutamic acid-to-alanine mutants than for the wild-type. The hyperphosphorylated state of the mutants may compensate for the increased amount of dimers sequestrated at transcriptionally inert sites apart from regulatory elements, where they do not contribute to gene activation. In sum, induction of genes with only a single GAS site in their promoters is largely unaffected by these mutations.

**Figure 7:**
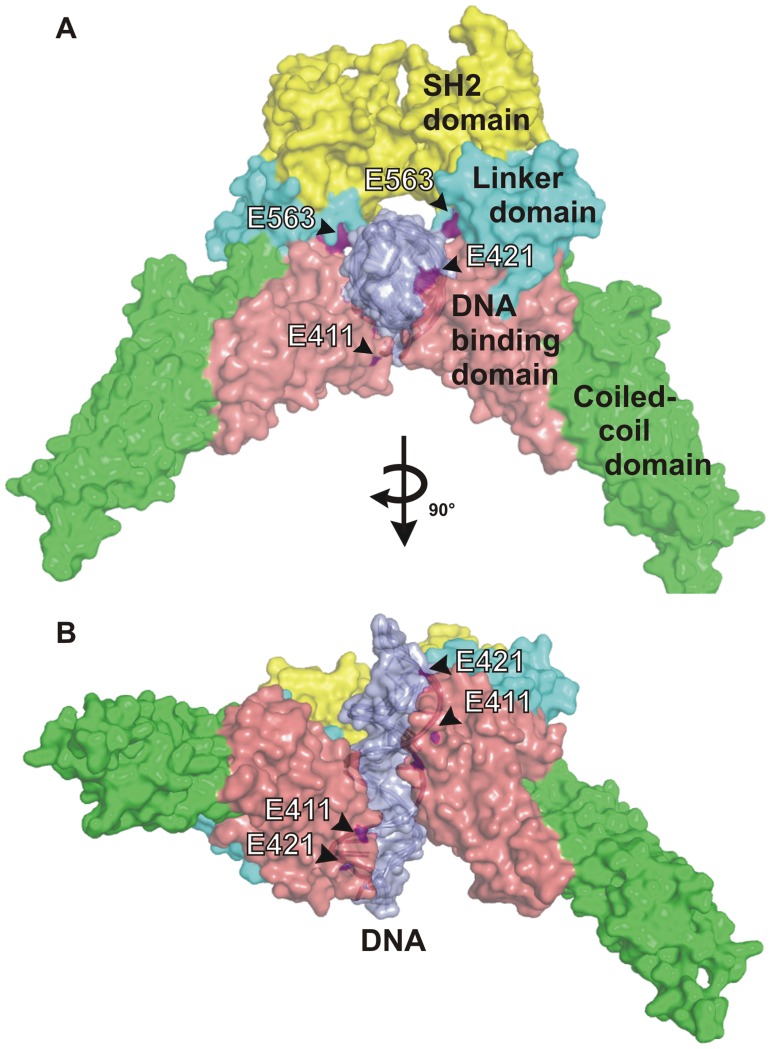
An array of surface-exposed glutamine acid residues aligns the STAT1 dimer to DNA. The model shows the localization of the residues (coloured in magenta) at the surface of a STAT1 dimer complexed to DNA viewed in axial (A) and frontal (B) orientation to the DNA double helix (indicated in grey).

The glutamic acid residues in the linker domain described here have similar functions to two other key residues (E411 and E421), which we have recently identified in the DNA-binding domain to be engaged in the release of dimeric STAT1 from DNA [43]. While E559 and E563 are positioned at the back in the carboxy-terminal core of the STAT1 dimer, the residues E411 and E421 are located anteriorly in relation to the DNA double helix. Our observations indicate that four distinct negatively charged residues on the surface of each monomer are all required for the dissociation from DNA and, furthermore, that mutation of only one of them has dramatic effects on the time course of STAT1 signalling. Thus, an array of eight glutamic acid residues, four on each monomer, appears to facilitate the correct alignment of the DNA, perpendicularly to the dyad symmetry axis of the parallel dimer conformation (Fig. 7).

In contrast to the glutamic acid residues that form a negatively charged abutment for the spatial orientation of the DNA molecule, the positively charged side chain of lysine 567 is required for high-affinity GAS binding. The exchange of this residue for alanine critically impedes GAS recognition and results in an almost complete loss of transcriptional activity, while neither IFNγ-induced nuclear accumulation nor binding to unspecific DNA is affected. In EMSA experiments, the K567A mutant shows weak or virtually no binding to a single GAS site, possibly because it also interacts with unlabelled duplex poly-deoxyinosine-deoxycytosine (poly-dIdC) present in the reactions as an unspecific competitor. However, dephosphorylation assays in the presence of DNA (Fig. 5C) as well as pull-down experiments with biotinylated oligonucleotides (Fig. 5D, E) both confirmed that K567A is not principally impaired in its contact to DNA, but has partially lost its sequence specificity for GAS sites. Although we found an insignificantly higher binding to 2xnon-GAS in pull-down assays as compared to the wild-type molecule (Fig. 5E), this appears not to affect the overall binding kinetics to unspecific DNA.

When a GAS motif is recognized by the immunoglobulin-like fold of the STAT1 DNA-binding domain, the terminal amino group of the lysine side chain presumably locks the DNA into a position that facilitates the transcriptional initiation. Upon binding to a GAS sequence, the helical structure of DNA appears to be slightly distorted by interactions between the terminal amino group of the K567 side chain and a phosphate residue in the DNA backbone. In the available crystal structure of DNA-bound STAT1, the electron density maps for the phosphate backbone adjacent to the K567 residue are not well resolved, suggesting that K567 interacts with the DNA in a way that transiently bends the DNA axis [44].

Up to now, it is unclear whether STATs find their GAS elements by walking along the axis of the DNA or by a hit-and-run mechanism [45]. Given the essential role of K567 in GAS recognition and the four glutamic acid residues in the alignment of dimeric STAT1 to DNA, our data are in line with a scanning mechanism of STAT dimers in their search for GAS elements. However, additional experiments are required to elucidate how precisely STAT1 can find their target sequences among the huge amount of unspecific genomic DNA.

## Supporting Information

Figure S1 Prolonged tyrosine phosphorylation of STAT1-E559A and -E563A in IFNγ-pretreated U3A cells in the absence of staurosporine inhibition. Cells were first stimulated for 45 min with 5 ng/ml IFNγ and then left untreated for 0, 5, 7 or 9 h, before tyrosine phosphorylation and protein expression of STAT1 was tested in cell extracts. A typical Western blot result (A) and a quantification of three similar experiments (B) are shown.
(TIF)
