# Supplementary figures and images for: A Conserved Motif in the Linker Domain of STAT1 Transcription Factor Is Required for Both Recognition and Release from High-Affinity DNA-Binding Sites
Source: PLoS One. 2014 May 21;9(5):e97633. doi: 10.1371/journal.pone.0097633 (PMC4029728; doi:10.1371/journal.pone.0097633)

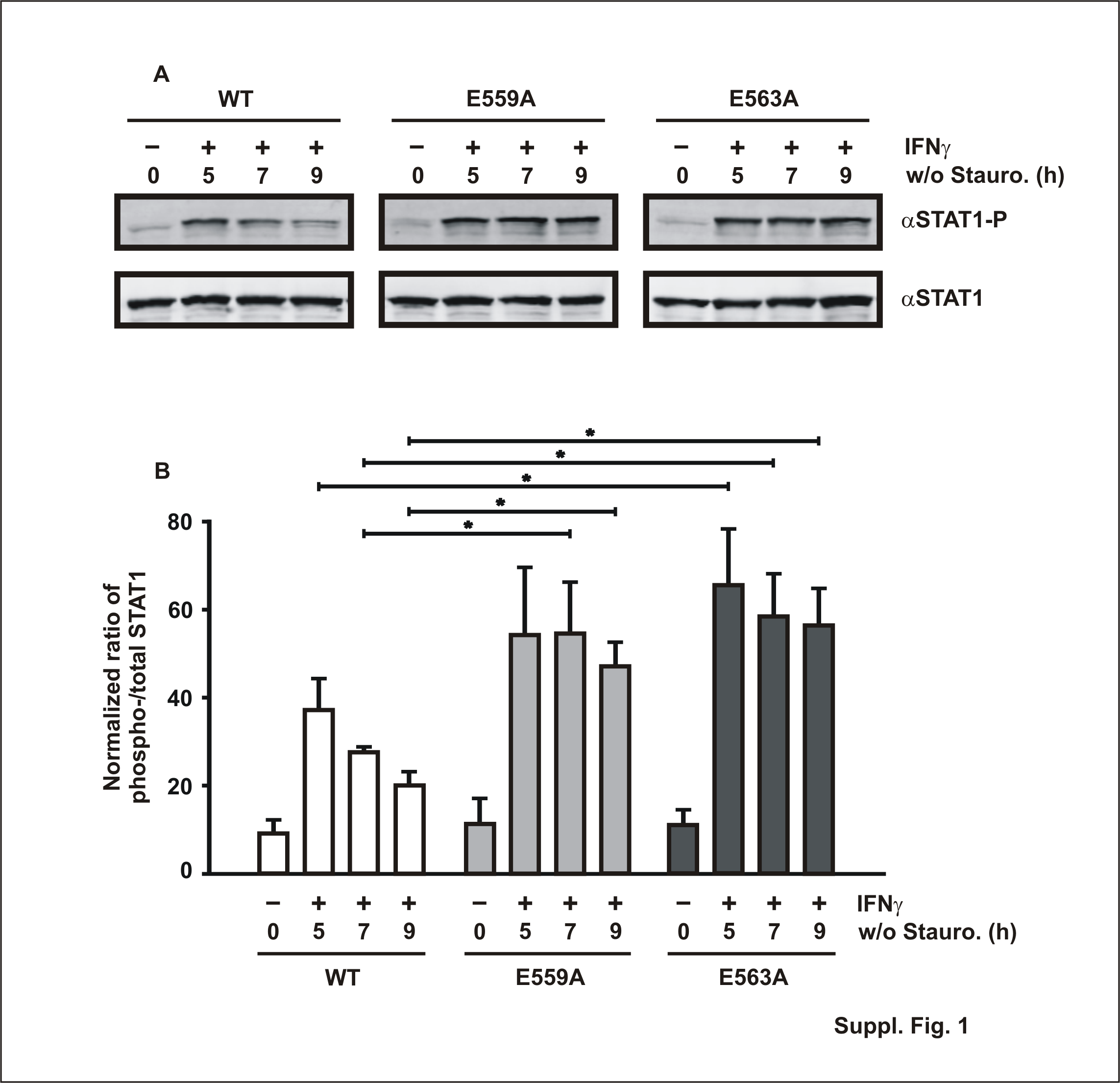

Supplement: Figure S1 — Prolonged tyrosine phosphorylation of STAT1-E559A and -E563A in IFNγ-pretreated U3A cells in the absence of staurosporine inhibition. Cells were first stimulated for 45 min with 5 ng/ml IFNγ and then left untreated for 0, 5, 7 or 9 h, before tyrosine phosphorylation and protein expression of STAT1 was tested in cell extracts. A typical Western blot result (A) and a quantification of three similar experiments (B) are shown. (TIF) [file pone.0097633.s001.tif]
